# *In vitro* and *in vivo* assessment of sporopollenin exine capsule preparations (SpECs) from *Lycopodium clavatum* spores

**DOI:** 10.1039/d5ra09999d

**Published:** 2026-04-07

**Authors:** Ammar Hasan, Jeremiah Stanley, Abu Saleh Md Moin, Hamna Begam, Sana Waris, Marah Abdulhadi, Khaled Greish, Andrew N. Boa, Manyam Praveen Kumar, G. Roshan Deen, Stephen L. Atkin, Alexandra E. Butler

**Affiliations:** a Research Department, School of Postgraduate Studies and Research, Royal College of Surgeons in Ireland, Medical University of Bahrain 228 Busaiteen 15503 Bahrain ahasan@rcsi.com rediffjerry@gmail.com amoin@rcsi.com hbegam@rcsi.com swaris@rcsi-mub.com pmanyam@rcsi.com satkin@rcsi.com aeb91011@gmail.com abutler@rcsi.com +97366760313; b Department of Molecular Medicine & Nanomedicine Unit, Al Jawhara Centre for Molecular Medicine, Genetics & Inherited Diseases, College of Medicine & Medical Sciences, Arabian Gulf University Manama Bahrain khaledfg@agu.edu.bh marahabdulhadi@hotmail.com; c Department of Chemistry, Hull University Hull UK a.n.boa@hull.ac.uk; d School of Medicine, Royal College of Surgeons in Ireland, Medical University of Bahrain 228 Busaiteen Bahrain rdeen@rcsi.com

## Abstract

This study investigated the potential allergenicity of sporopollenin exine capsule preparations (SpECs) derived from plant pollen or spores as allergenicity could limit their use as a therapeutic oral drug delivery system. As allergenicity may differ depending on the method of preparation, raw spores from *Lycopodium clavatum,* together with 6 *L. clavatum* SpEC preparations were evaluated *in vitro* by adenosine triphosphate (ATP) bioassay for cell viability. Subsequently, *in vivo* evaluations were performed in 6–8 week male Balb/C mice gavaged daily with raw *L. clavatum* spores, a negative control (PBS), or one of SpEC-3, 4 or 5 preparations for five consecutive days; gastrointestinal tissues were collected 6 hours following the final gavage. *In vitro* cytotoxicity studies showed that, compared to the cell control, *L. clavatum* spores and SpEC-2 and SpEC-4 preparations showed significantly decreased cell viability, while no significant differences in cell viability were found for SpEC-1, 3, 5 or 6 preparations, all of which were extracted more intensively than SpEC-2 or SpEC-4 preparations. *In vivo* safety studies showed no increase in CD68-positive macrophage cell infiltration in any region of the gastrointestinal tract (stomach, duodenum, jejunum, ileum or colon), liver or kidneys. No exines were found in any tissue. We concluded that, whilst differing SpEC manufacturing processes may have residual *in vitro* cytotoxicity, this did not translate to an acute *in vivo* oral gastrointestinal immune response, suggesting their safety as a vehicle for gastrointestinal pharmaceutical delivery.

## Abstract 2

This study investigated the potential allergenicity of sporopollenin exine capsule preparations (SpECs) derived from plant pollen or spores as allergenicity could limit their use as a therapeutic oral drug delivery system.

## Graphical abstract

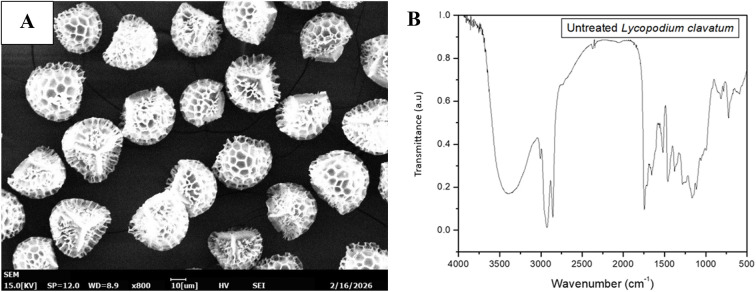

## Introduction

The unique properties of plant pollen and spores have translated into a large body of research exploring their potential applications. The use of pollen can be either due to the bioactive compounds extracted from pollen, which possess medicinal properties, or due to the structural characteristics of pollen grains themselves, which can be leveraged for biomedical purposes. Pollen-derived bioactive compounds have demonstrated a wide range of health benefits, including antioxidant, anti-inflammatory, and antimicrobial activities. These compounds can be isolated and utilized in various pharmaceutical preparations and nutraceutical products. Furthermore, the complex structure of pollen grains, including their strong outer walls and internal compartments, has inspired their use as natural biomaterials. Pollen-based scaffolds can be used in tissue engineering to promote cell growth and regeneration, further, they can be employed as drug delivery systems to target specific tissues or organs.^1–5^ Other applications for indwelling or *in vivo* pollen structures include the use of a cryogel for hemorrhage control^6^ and pollen-based magnetic microrobots for cancer therapy^7^ and there is evidence that the body has protective mechanisms to degrade pollen that may enter the bloodstream.^8^ However, for any application that requires an *in vivo* application, either as an indwelling device in tissue or as a circulating entity within the bloodstream, it is critical to know the potential that the device has to cause harm and *in vitro* testing of the material is both economically and ethically the first logical step to determine safety.

Sporopollenin is the material that pollen and spores are composed of for their structural integrity.^9,10^ Sporopollenin exine capsules (SpECs) are extracted from either pollen or plant spores in the form of empty porous microcapsules penetrated by nano-diameter sized channels.^11,12^ These channels enable a variety of active ingredients (AIs) to be loaded into the SpEC chambers; hence, giving universality to the system to encapsulate such as a variety of antibody fragments, proteins and peptides.^11–13^ Controlled release can be aided by the use of excipients co-encapsulated either with the AI or that surround the SpECs. The topography and size of a SpEC are characteristic of the plant species from which it is derived. This allows for the selection of a monodisperse delivery vehicle with high uniform dimensions. This depends on the species chosen and the sizes typically range from 5 to 250 µm. Importantly, the well-defined topological features [*e.g. Helianthus annuus* SpECs have long spikes and *Lycopodium clavatum* have serrated coronets^6,10^] that may contribute to the SpECs having bioadhesive and enhanced bioavailability properties following oral ingestion.^14,15^ SpECs are composed of sporopollenin, which is a highly cross-linked lipid-based polymer derived principally from fatty acids; it is devoid of proteins but possesses accessible phenolic groups with antioxidant activity^16^ and carboxylic acid functional groups which offer modification of the polarity and good bioadhesive properties.^10,17–19^ The cross-linking in sporopollenin endows it with pronounced physicochemical stability. It is stable against stomach acid, intestinal alkali and a wide range of digestive enzymes. It is also stable against a wide range of organic and inorganic solvents. In addition, the SpECs have good elasticity without breakage, which enables slow release of an AI due to shear over surfaces such as skin and intestinal wall. SpECs are protein-free and are non-toxic and non-allergenic.^17^

*Lycopodium clavatum* (*L. clavatum*) spores were selected as a model source of sporopollenin exine capsules due to their commercial availability, uniform size distribution, structural robustness, and extensive prior use in encapsulation studies. Their well-characterized morphology and reproducible processing profile make them particularly suitable for systematically evaluating the impact of chemical treatments on residual surface biomolecules and biological responses.

**Fig. 1:**
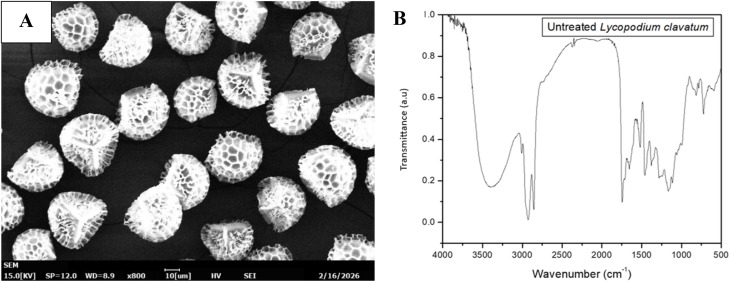
(A) Scanning electron micrograph of *Lycopodium clavatum*, (B) FTIR of *Lycopodium clavatum*. Sporopollenin is defined as the principal component of the outer exine layer of pollen and spores and as the chemically resistant residue remaining after acetolysis.^11^ The structure of sporopollenin, particularly in *Lycopodium clavatum*, has been reported to consist of two major building units that together form the exine framework.^20^ The first comprises a macrocyclic oligomeric and/or polymeric backbone composed of polyhydroxylated tetraketide-like monomeric units, which provide structural rigidity to the biopolymer. The second component is a poly(hydroxy acid) network in which the terminal hydroxyl groups are covalently linked *via* ether bonds to the hydroxylated macrocyclic backbone, yielding a highly crosslinked sporopollenin biopolymer with dendrimer-like architecture.^20^. (B) The FTIR spectrum of untreated *Lycopodium clavatum* spores shows a broad absorption band at 3400–3300 cm^−1^ attributed to O–H stretching vibrations of hydroxyl groups and adsorbed moisture. Strong bands at 2925 and 2854 cm^−1^ correspond to asymmetric and symmetric stretching of aliphatic CH_2_ groups, indicating the presence of long-chain aliphatic structures. A prominent absorption at ∼1735 cm^−1^ is assigned to ester carbonyl (C=O) stretching vibrations. The bands at ∼1650 and ∼1540 cm^−1^ are attributed to amide I and amide II vibrations, respectively, confirming the presence of proteinaceous material. Multiple peaks in the 1200–1000 cm^−1^ region arise from C–O and C–O–C stretching vibrations characteristic of sporopollenin and polysaccharide structures.

All the particles from a single species of plant have almost the same size and decorations such as for *L. clavatum* particles, used in this study that are 28 µm in diameter and take a retusoid trilete shape and are monodispersed, as shown in Fig. 1A.

The particle surface architecture is randomly pierced by nanopores of *ca.* 40 nm diameter, allowing material to enter/exit the spore particle.^10^ The SpECs are elastic and pressure results in partial release of an encapsulated active agent, with 70–80% emptying occurring after a series of pressings,^11^ and show resilience to both alkalis and acids, and the ability to withstand temperatures up to 250 °C.^11^

The presence of aromatic moieties in sporopollenin remains debated. Although earlier degradation studies suggested phenolic constituents, more recent spectroscopic analyses indicate that the polymer is predominantly aliphatic and exhibits little to no extensive aromatic character.^20^ Nevertheless, oxidative electrochemical studies suggest that its redox activity may arise from conjugated phenolic or related functionalities within the polymer, giving rise to either a two-electron, two-proton process or a two-electron, one-proton process.^16^ Investigations of charge transport across sporopollenin particles further indicate rapid surface electron transfer, which may account for the observed antioxidant behaviour through charge delocalisation within this semi-conducting biopolymer.^16^ Recent state-of-the-art solid-state NMR and thioacidolysis studies indicate that sporopollenin is primarily composed of aliphatic polyketide-derived polyvinyl alcohol-like units and 7-*O-p*-coumaroylated C16 aliphatic chains, crosslinked through dioxane (acetal) moieties.^20^

Raw and refined pollen or spores are recognised as having allergenic properties, due to the presence of proteins and protein residuals, contained within the pollen/spore shell and on its surface.^21^ The shell constitutes two layers, the outer highly resilient layer (exine) composed of sporopollenin and the inner (intine) layer composed mainly of cellulose.^22^ Removing all the cytoplasmic content that will include the protein, lipids, carbohydrate and genetic material is therefore crucial prior to any potential clinical application. The readily available, relatively large inner cavity of the empty SpECs after extracting the cytoplasmic proteins, have a uniformity of size and morphology that makes them a suitable candidate for controlled delivery of pharmaceutical drugs^9,17^ SpECs have been employed as an innovative drug delivery system,^23,24^ filling them with an active substance^12^ and subsequently releasing the active substance through the same nano-channels that were initially used to load the SpECs.^22,25^ Enhanced bioavailability orally has been shown for omega oil and vitamin D loaded SpECs.^14,26^

One common concern is the cytotoxicity of the prepared SpECs that may limit their use as a drug delivery vehicle and whether different manufacturing methods may mitigate any potential immune response. To the best of our knowledge, there are no data in the scientific literature on the relative allergenicity of chemically treated *Lycopodium clavatum*, nor assays to determine the allergenicity, other than direct expensive animal testing. Therefore, this study, the first of its kind, was undertaken with the assessment of *in vitro* cytotoxicity of differing SpEC manufacturing methods, followed by the immunogenic assessment of an acute *in vivo* oral challenge response.

## Materials and methods

### Materials

*Lycopodium clavatum* spores were obtained from Sporomex Ltd (Driffield, UK). Sodium hydroxide (NaOH), sodium hypochlorite (NaOCl), sodium bromide (NaBr), sodium dodecyl sulphate (SDS), phosphorous pentoxide (P_2_O_5_), hydrogen peroxide (H_2_O_2_), hydrochloric acid, ethanol and acetone were purchased from Sigma-Aldrich and used as received.

### Preparation of exines by various chemical treatments

**Fig. 2:**
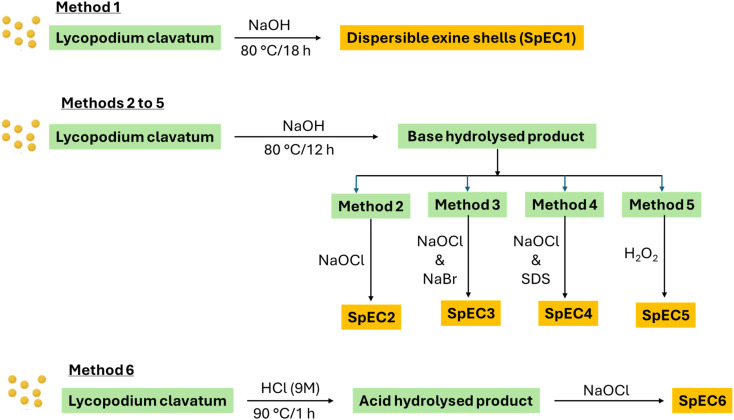
Schematic illustration of the various chemical processes in the preparation of SpECs.

The *L. clavatum* was subjected to various chemical treatments as illustrated in Fig. 2.

#### Method 1: preparation of dispersible exine shells (DES)

Raw *L. clavatum* spores (500 g) were stirred in 6% (w/v) NaOH aqueous solution (2.8 L) for 16 h at 71 °C (internal) and 80 °C (external). The product was filtered under vacuum and washed with hot water (60–70 °C) and then with cold water (*ca.* 20 °C), until the filtrate became colorless and neutral pH. The product was dried at 60 °C overnight. Yield: 200 g (45%). Elemental analysis (%): C: 60.13, H: 8.21, N: 0. Sample code: SpEC1.

#### Method 2: base hydrolysis and hypochlorite bleaching

Raw *L. clavatum* spores (200 g) was stirred in 6% (w/v) NaOH aqueous solution (900 mL) at 80 °C for 6 h, followed by filtration and washing with hot water (2 × 500 mL). The product was resuspended in 6% (w/v) NaOH aqueous solution (900 mL) and stirred at 80 °C for another 6 h, followed by filtration. The solid product was washed first with hot water (6 × 500 mL) and then with ethanol (2 × 200 mL). The solid was dried under vacuum over phosphoros pentoxide to constant weight. Yield of base hydrolysed product (BHS): 40%. The dried solid was bleached with sodium hypochlorite as follows: the base hydrolysed product (3 g) was stirred in 50 mL aqueous sodium hypochlorite (7% w/v) at 50 °C for 1 h. The bleached product was recovered by filtration (porosity grade 3) and washed sequentially with hot water (3 × 50 mL) and ethanol (3 × 50 mL). The product was dried under P_2_O_5_. Yield: 84%. Elemental analysis (%): C: 61.65, H: 9.32, N: 0.08. Sample code: SpEC2.

#### Method 3: base hydrolysis and sodium hypochlorite bleaching with sodium bromide

BHS from method 2 (3 g) was stirred in 50 mL solution of sodium hypochlorite (7%) and sodium bromide (60 mg) at 50 °C for 1 h. The product was filtered (porosity grade 3) and washed sequentially with hot water (3 × 50 mL) and ethanol (3 × 50 mL). The solid was dried over P_2_O_5_. Yield: 89%. Elemental analysis (%): C: 53.8, H: 7.22, N: 0. Sample code: SpEC3.

#### Method 4: base hydrolysis and sodium hypochlorite bleaching with sodium dodecyl sulphate

BHS from method 2 (3 g) was stirred aqueous sodium hypochlorite (7%) and SDS (0.50 g) at 50 °C for 1 h. The product was filtered (porosity grade 3) and washed sequentially with hot water (3 × 50 mL) and ethanol (3 × 50 mL). The solid was dried over P_2_O_5_. Yield: 84%. Elemental analysis (%): C: 52.60, H: 7.01, N: 0. Sample code: SpEC4.

#### Method 5: base hydrolysis and hydrogen peroxide bleaching

BHS from method 2 (10 g) was stirred in 100 mL of 30% (w/w) H_2_O_2_. The pH of the suspension was adjusted to between 10–11 with 2 M NaOH and heated at 78 °C for 1 h. The product was filtered (porosity grade 3) and washed sequentially with hot water (3 × 80 mL) and ethanol (3 × 80 mL) and dried over P_2_O_5_ under vacuum. Yield: 85%. Elemental analysis (%): C: 53.21, H: 7.11, N: 0. Sample code: SpEc5.

#### Method 6: acid hydrolysis and sodium hypochlorite bleaching

Raw *L. clavatum* (100 g) was suspended in 450 mL of 9 M HCl and heated at 90 °C for 1 h. The suspension was filtered, and the isolated product was washed with water (3 × 600 mL) until the filtrate was neutral and further washed with ethanol (2 × 200 mL). The solid product was dried to constant weight under vacuum over P_2_O_5_. Elemental analysis: C: 67.97%, H: 9.19%, N: 0%. The dried solid was then bleached with sodium hypochlorite (7% w/v, 50 mL) for 1 h at 50 °C. The suspension was filtered (porosity grade 3) and washed sequentially with water (3 × 50 mL) and ethanol (3 × 50 mL) and dried over P_2_O_5_ to constant weight. Elemental analysis (%): C: 60.07, H: 8.99, N: 0. Sample code: SpEC6.

### Fourier-transform infra-red (FTIR) spectroscopy

FTIR spectra of the SpEC samples were recorded in the wave number range of 4000–500 cm^−1^ with a spectral resolution of 0.5 cm^−1^, using a Bruker Alpha-FT-IR spectrophotometer (Ettlingen, Germany). Samples were mixed with dry potassium bromide (spectroscopic grade) and made into a thin transparent disc.

### Scanning electron microscopy

The SpEC samples were imaged on a scanning electron microscope (Invenso SEMoscope, Cambridge, MA, USA) with an accelerating voltage of 6–20 kV. The samples were sputtered with gold (10 nm layer) for 2 min on a Inovenso SPT-20 sputter (Cambridge, MA, USA) operating at a voltage of 15 kV.

### *In vitro* studies

#### Cell culture

Human epithelial BEAS-2B cells (ATCC CRL-3588, Virginia, USA), a cell line used typically in toxicological studies, were revived as per ATCC recommendation. The cells were maintained by serial passage in DMEM (Sigma D5796, Darmstadt, Germany) with 10% fetal calf serum (Gibco 10270-106 New York, USA) and 1% penicillin–streptomycin (Sigma P4333, Darmstadt, Germany) in Nunclon Delta T75 flasks (ThermoFisher 156499 Massachusetts, USA). Cells were subcultured every third day before they reached confluent growth and terminal differentiation.

#### SpEC and *L. clavatum* spore suspensions

SpEC preparations (SpECs 1–6) and raw intact *L. clavatum* spores available in dry powder form were weighed and reconstituted in sterile PBS (Gibco 10010023 New York, USA) to a concentration of 10 mg mL^−1^. The preparations were kept on shaker and rocked gently overnight to ensure soakage and even dispersion of the SpEC preparations and spores. Overnight soaked preparations settled to the bottom of the solution. The preparations were then sterilized by autoclaving in 121 °C at 15 psi for 15 minutes and stored in room temperature until further use.

#### Adenosine triphosphate (ATP) assay for cell viability

2 × 10^4^ BEAS-2B cells were seeded per well in a 96 well flat bottom white plate (Corning 3917 New York, USA) and incubated at 37 °C overnight to obtain a confluent monolayer mimicking the epithelial surface. Each of the SpEC/*L. clavatum* spore preparations were dispersed by pulse vortexing and were added to corresponding wells to achieve a concentration of 1 mg mL^−1^ in the well. Silica gel granules (Sigma 717185 Darmstadt, Germany) at the same concentration and an untreated cell control were used as negative controls. The plate was incubated at 37 °C overnight to allow the test substances to settle on the cell monolayer with 100% coverage. On the next day (48 hours), the supernatant culture medium was removed by aspiration and the exines were washed off the surface of the cell monolayer using warm PBS. The cell monolayer was treated with the ATP Determination Kit (ThermoFisher A22066 Massachusetts, USA) as per the manufacturer's instructions. Luminescence was read in CLARIOstar Plus (BMG Labtech, Ortenberg, Germany) with the inbuilt QUICK LUM protocol. A standard curve was plotted, and the luminescence signals were translated to ATP concentrations in picomoles. Each sample was tested in triplicate and the mean value was taken as the final ATP concentration.

### *In vivo* studies

#### Animals

Male Balb/C strain mice (*Mus musculus*) were bred in the animal facility at Arabian Gulf University, Bahrain. All animal protocols were approved by the Ethical Committee for Animal Experiments of Arabian Gulf University (reference number E29-PI-2-23).

#### Housing conditions

6–8 week old mice were maintained in plastic cages under specific pathogen-free (SPF) conditions. Standard cages (33 × 15 × 13 cm), containing 2 mice per cage, were cleaned daily. Mice were observed daily and weighed every 2 days. All mice were maintained on a 12 h light/dark cycle and fed standard chow (fat 4.2%, protein 20%, carbohydrates 55.3%, fiber 4%, other 16.5%) *ad libitum*. Water was available *ad libitum*. All tests were performed during the light cycle.

#### SpEC preparations

Raw *L. clavatum* spores, SpEC-3, SpEC-4, and SpEC-5 preparations were administered by oral gavage *in vivo*. Gavage with PBS served as the negative control. SpEC-4 preparation was selected due to its reduced cell viability in the *in vitro* cytotoxicity studies, whereas SpEC-3 and SpEC-5 were chosen as representative of preparations that showed no significant difference in cell viability compared to the control in those same studies. The raw *L. clavatum* spores and SpEC preparations were administered orally at a concentration of 1.4 mg suspended in 150 µl of phosphate buffered saline (PBS). The negative control group received PBS orally. A total of 30 mice were utilized (*n* = 6 per group).

#### Oral gavage

Gavage needles were obtained from ShinMedico (ShinMedico, Japan). Oral gavaging (1.4 mg of the designated exine mixed with 150 µl of PBS. The exact volume of PBS was calculated depending upon individual mouse weight; the total administrated did not exceed 10 µl g^−1^) was conducted on all mice at 10:00 am daily for five consecutive days by an experienced veterinarian (AH). Tissues were collected six hours after the final gavage.

#### Sacrifice

Mice were sacrificed using carbon dioxide inhalation. The abdominal cavity was immediately opened, and the entire gastrointestinal tract (GIT) retrieved and divided into the following anatomical sections: stomach, duodenum, jejunum, ileum and colon. Liver and kidney were also harvested. The tissue was fixed overnight in 10% formaldehyde and, the following day, processed and embedded in paraffin.

### Processing and analysis

Blood was collected in EDTA at sacrifice and a full blood count for white blood cells, lymphocytes, monocytes and neutrophils were determined using a Beckman Coulter counter (Beckman Coulter, Brea, USA).

#### Tissue preparation and immunohistochemistry staining

One section (4–5 µm) from each block was stained for hematoxylin/eosin (H&E). Additional tissue sections (4–5 µm) underwent pretreatment using the Leica BondMax platform (Leica Biosystems, Deer Park, Illinois, USA). Subsequently, the sections were incubated with primary antibody, CD68 (ThermoFisher Scientific, Waltham, MA, USA; cat. # MA5-13324; concentration 1:200 overnight) and then treated with the detection system BOND Polymer Refine Detection (Leica Biosystems). Quality control (QC) was performed to ensure proper staining and outcomes and documented in the QC log.

#### Cell counting

Sections of GIT tissue were viewed using a KFBIO Digital Pathology microscope and scanner (KFBIO, Yuyao Technology Innovation Center, ZheJiang, P.R.C https://www.kfbiopathology.com/); brightfield images acquired using a 20× objective lens. Images were viewed using K-Viewer (Novelsis Technology Solutions, Halkapinar, Konak-Izmir, Turkey https://novelsis.com/k-viewer-en/), and cells were counted using 10× magnification. Areas of 90 000 µm^2^ in size from comparable anatomical regions of the GIT were chosen for the counting of positive CD68 cells; 10 areas each from stomach, ileum and colon, and 20 areas each from duodenum and jejunum. Cell counts were conducted automatically using Image J software (https://imagej.net/ij/). The accuracy of the automated counting was validated through manual recounting by three blinded referees (AH, HB and AM). Numbers of CD68-positive cells per mm^2^ tissue were quantified for each region of the GIT within each test group and compared with the corresponding tissues from the negative control group.

### Statistical analysis

#### In vitro

For the *in vitro* assay of ATP reduction reflecting cytotoxicity, each test sample was compared against the negative control silica gel. Statistical significance was ascertained in Excel using the two-tailed unpaired Student's *t* test.

#### In vivo

For analysis of the histological sections, numbers of CD68-positive cells per mm^2^ tissue were quantified for each region of the GIT within each test group and compared with the corresponding tissues from the negative control group using the two tailed unpaired Student's *t* test.

## Results

### Sporopollenin exine capsule (SpEC) and analysis

**Fig. 3:**
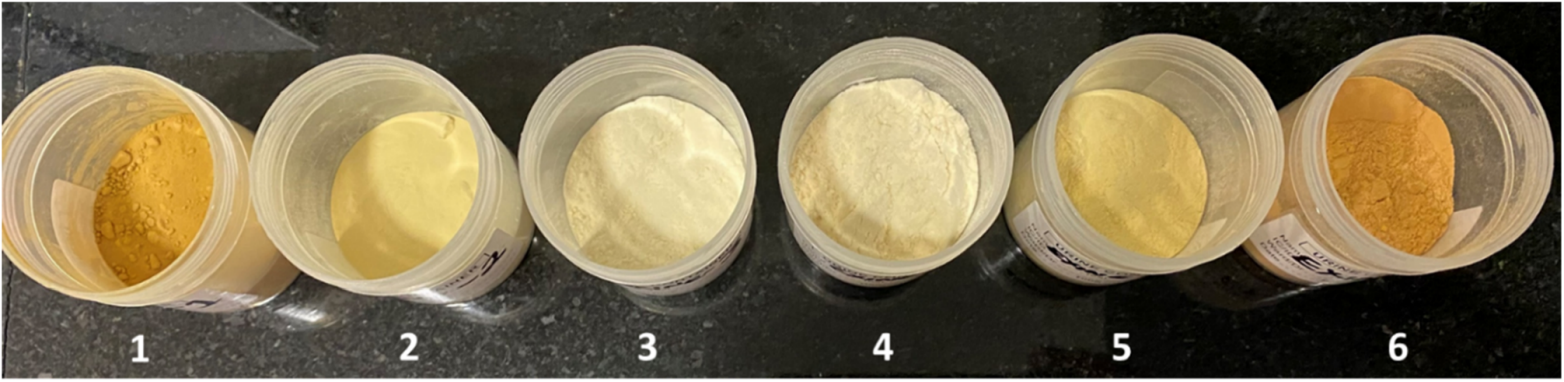
Appearance of SpECs after various chemical treatment process.

The physical appearance of SpECs after various chemical treatment processes is shown in Fig. 3. The different colour contrast exhibited is attributed to carbonisation and the removal of various chemical components by alkaline and acid hydrolysis, oxidation and bleaching. The combustion elemental analysis of SpEC 1 to SpEC 6 showed 0% nitrogen content, which indicates that almost all detectable amount of the proteinaceous substances and intine have been removed from the raw pollen by the various chemical treatments. The elemental analysis of raw *L. clavatum* showed 0.74% nitrogen content which arises from various components such as amino acids, proteins, alkaloids and nucleic acids. The elemental analysis is often used as a purity indicator in the preparation of exines. Furthermore, the results indicate that the cleaning process with acids and bases is complete and all the internal proteins inside the shells have been removed.

**Fig. 4:**
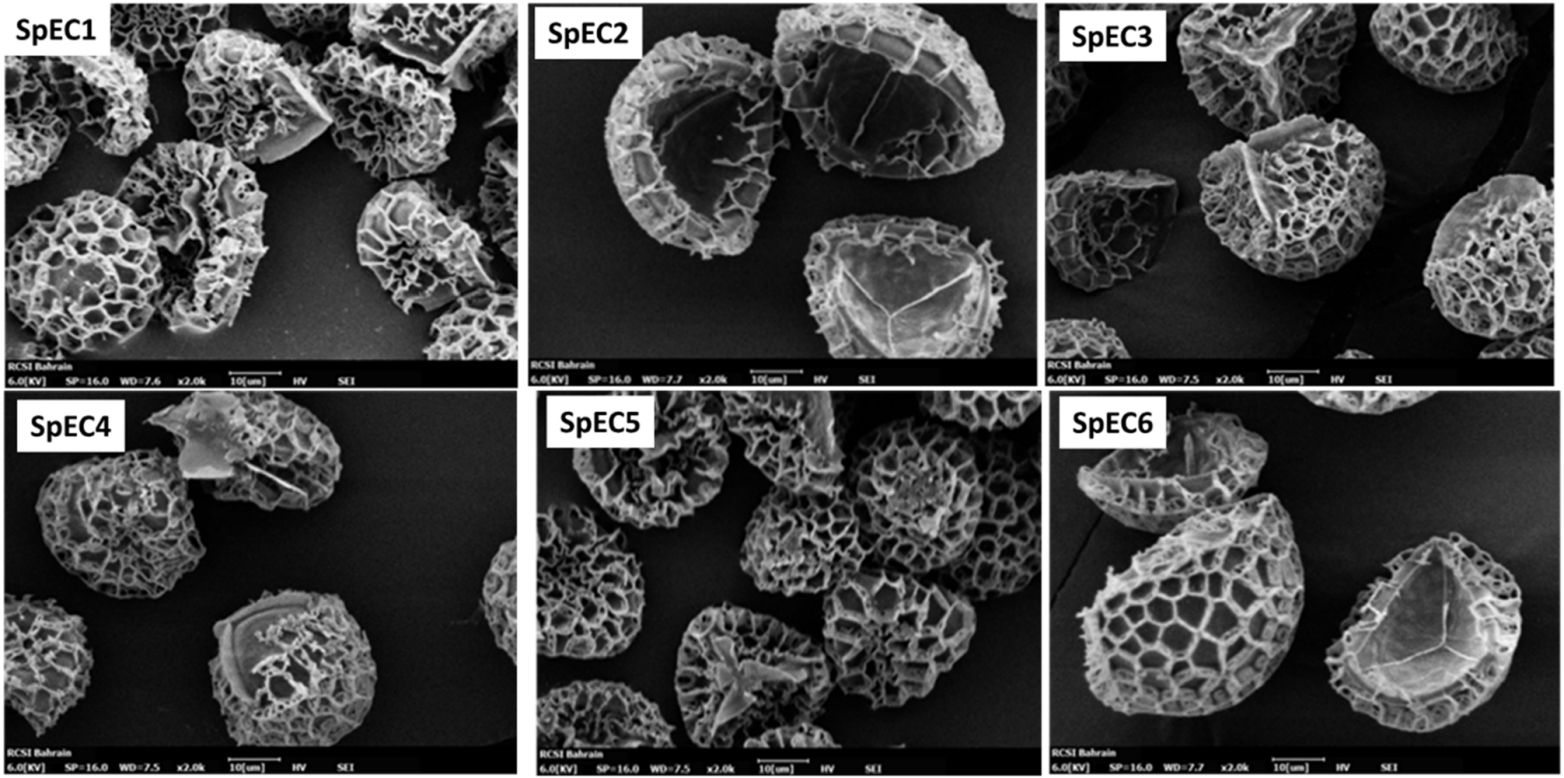
Scanning electron micrographs of various SpECs. The trilite scar is clearly visible. Some spores appear buckled due to the vacuum created during SEM measurement.

Scanning electron micrographs of the SpECs are shown in Fig. 4. The characteristic reticulate ornamentation of *L. clavtum* is clearly observed for all the samples indicating that the structural features are preserved despite the various chemical treatments. The average size range of the SpECs is 27–32 µm. The SpECs appear clean and no debris is observed, further the particles appear sucked in due to the vacuum employed during SEM indirectly indicating that the particles are hollow with the removal of the intine layer after the chemical treatment.

**Fig. 5:**
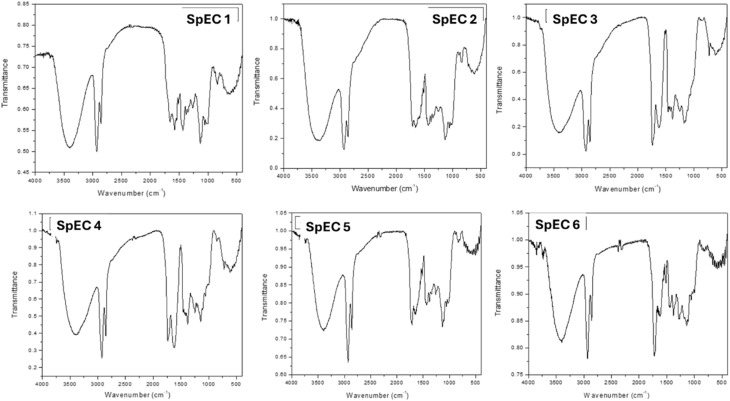
Fourier-transform infra-red (FTIR) spectroscopy spectra of SpECs.

The FTIR spectra of the SpECs are shown in Fig. 5. The strong and sharp peaks in the range 3500–3400 cm^−1^, and 2930–2857 cm^−1^ for all the SpECs indicate the presence of hydroxyl (–OH) and methylene (CH_2_) and methyl (CH_3_) functional groups.^27^

Sharp peaks at 1683 cm^−1^ and 1588 cm^−1^ are due to ketone and carboxyl functional groups (SpEC 1 and SpEC 2). The ketone functional group at 1750 cm^−1^ is more prominent for SpEC 3, SpEC 4, SpEC 5 and SpEc 6. Other peaks in the range 1600–1500 cm^−1^, 1200–1000 cm^−1^ and 840 cm^−1^ are due to aliphatic carbons, ethers and aromatic C–H groups. The peaks around 1500 cm^−1^ for the samples are attributed to sporopollenin aromatic and CH bending modes.^28^ The FTIR results further confirm the removal of organic substances and preservation of sporopollenin structures. Based on the various chemical treatment processes, SEM, and FTIR characterizations, SpEC 4 is expected to be the most highly processed due to surfactant-based solubilisation removal of pigments and other organic compounds.

Overall, the chemical treatments primarily modified surface oxygen-containing functional groups (hydroxyl and carbonyl moieties) while preserving the aliphatic and aromatic backbone characteristic of sporopollenin. Oxidative treatments (NaOCl and H_2_O_2_ systems) increased carbonyl functionality, whereas alkaline treatment promoted hydrolysis and surface cleaning. The complete absence of nitrogen across all samples confirms the successful removal of protein residues and supports the chemical robustness of the sporopollenin framework.

**Fig. 6:**
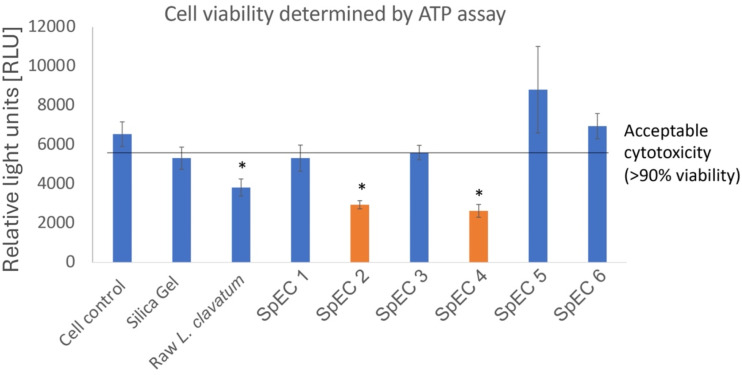
*In vitro* cell cytotoxicity results for raw *L. clavatum* spores and the 6 SpEC preparations. Relative to cell control, there was a significant decrease in cell viability for the raw *L. clavatum* spores and the SpEC 2 and SpEC 4 preparations. By contrast, relative to the cell control, there was no significant difference in cytotoxicity for the SpEC 1, 3, 5 or 6 preparations.

The *in vitro* cytotoxicity data indicate that the reduction in cell viability observed for raw *L. clavatum* spores and the SpEC 2 and SpEC 4 preparations is primarily attributable to surface-associated biomolecules rather than the sporopollenin exine itself. The significant decreases in viability (42%, 55%, and 60%, respectively) suggest that untreated or partially processed spores retain bioactive components capable of eliciting adverse cellular responses (Fig. 6). Native spores possess proteins, glycoproteins, and carbohydrate-rich macromolecules embedded within or adsorbed onto the outer exine layer, which may act as immunogenic determinants. These surface carbohydrates can function as pathogen-associated molecular patterns (PAMPs), interacting with pattern recognition receptors such as toll-like receptors or lectin-type receptors on mammalian cells, thereby triggering inflammatory signaling cascades, oxidative stress, and downstream reductions in metabolic activity.

In contrast, the SpEC 1, 3, 5, and 6 preparations exhibited no significant cytotoxicity, with minimal or no reduction in cell viability. Notably, the NaOH/H_2_O_2_ (SpEC 5) and HCl/NaOCl (SpEC 6) treatments are chemically rigorous and specifically target glycosidic linkages and proteinaceous residues within the outer spore layer (Fig. 6). These processes hydrolyze carbohydrate moieties, oxidatively degrade residual biomolecules, and effectively strip immunogenic surface constituents, leaving behind the highly cross-linked and chemically inert sporopollenin framework. The absence of cytotoxicity following these treatments therefore supports a carbohydrate-mediated mechanism of cellular recognition.^29^ Collectively, these findings indicate that cytotoxic effects are governed by the biochemical composition of the spore surface rather than the sporopollenin exine structure itself, and that appropriate chemical processing renders the exine capsules biologically inert and suitable for biomedical applications.

#### In vivo

The 1.4 mg dose per mouse is equivalent to a 4 g dose for a 70 kg person that is four times higher than the oral studies done in humans^14,15^ and for a longer duration; therefore, this likely represents the highest toxicological dose in humans, hence a single concentration was used. From the gavage safety studies in the mice, there were no phenotypic or behavioral abnormalities in the mice and no changes in feeding or weight during the course of the study. There were no differences between groups for white blood cells, lymphocytes, monocytes or neutrophils (*p* > 0.05) as shown in Table 1.

**Table 1:** White blood cell count (WBC; normal range 6.0–15.0), lymphocyte count (LYM; normal range 3.4–7.44), monocyte count (Mon; normal range 0.0–0.6) and neutrophil count (Neu; 0.5–3.8) for each group at sacrifice (5 days) that did not differ between groups and were all in the normal laboratory reference range

Group	WBC (10^9^ L^−1^)	LYM (10^9^ L^−1^)	Mon (10^9^ L^−1^)	Neu (10^9^ L^−1^)
SpEC1	7.51 ± 0.66	5.35 ± 1.32	0.51 ± 0.12	1.65 ± 0.59
SpEC2	7.73 ± 0.95	5.62 ± 0.35	0.22 ± 0.27	2.95 ± 0.86
SpEC3	4.84 ± 1.53	2.98 ± 0.73	0.23 ± 0.12	1.63 ± 0.89
SpEC4	4.55 ± 1.73	2.45 ± 1.58	0.24 ± 0.17	1.87 ± 0.44
SpEC5	6.24 ± 3.42	4.23 ± 2.41	0.2 ± 0.18	1.82 ± 0.99
SpEC6	5.28 ± 0.6	2.54 ± 0.57	0.21 ± 0.09	0.53 ± 0.12

**Fig. 7:**
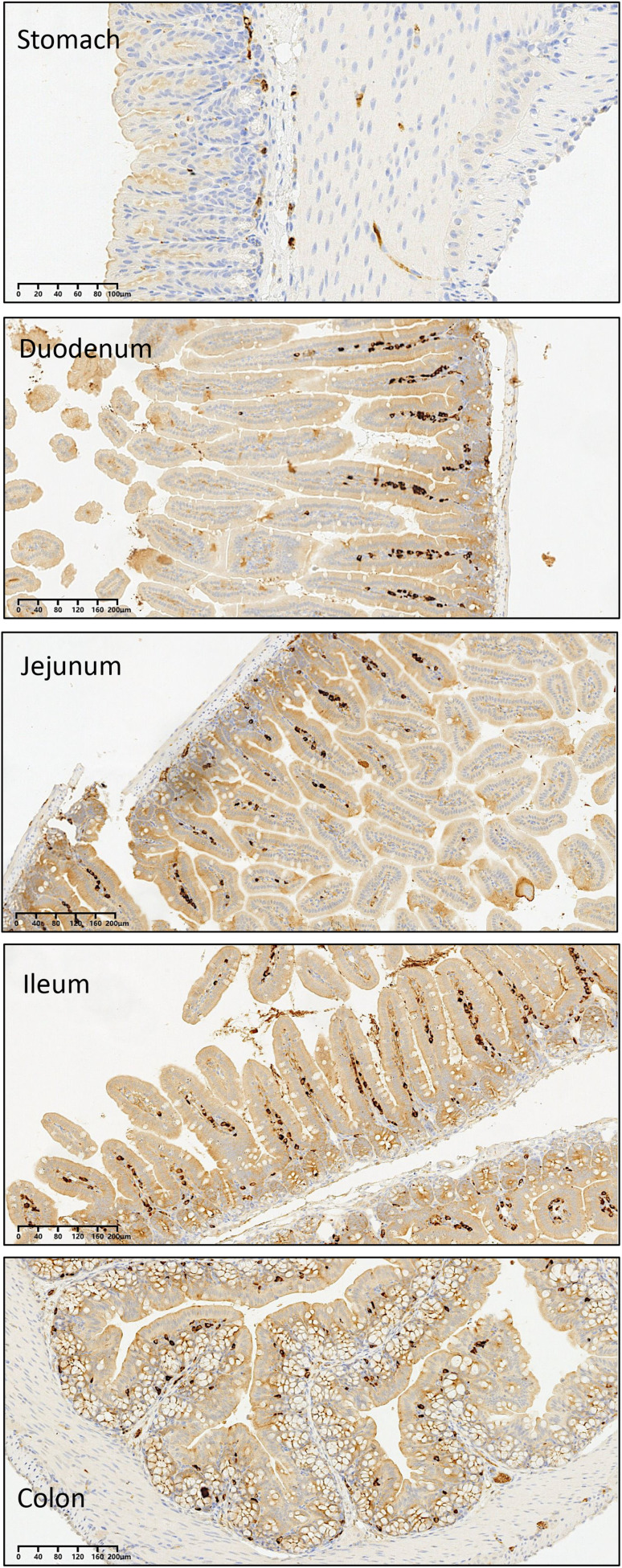
Representative sections from the gastrointestinal tract (GIT) of mice stained for CD68 as a macrophage marker. No differences in the number of macrophages present in each region of the GIT [stomach, small intestine (duodenum, jejunum and ileum) and colon], were found between any of the groups (raw *L. clavatum* spores and SpEC formulations 1, 2, 3, 4, 5 and 6). Images acquired at 10× magnification. Control sections indicating no indicative damage of liver and kidney are shown in SI Fig. 1.

As anticipated, CD68 positivity was seen in all sections from all regions of the GIT in all mice due to the resident macrophage population. However, there was no increase in CD68-positive macrophage cell infiltration, nor any changes in their tissue distribution, in any of the mice regardless of their treatment with raw *L. clavatum* spores, SpEC-3, SpEC-4 or SpEC-5 preparations compared with the negative PBS control. This indicates that there was no inflammation or cell damage in any region of the GIT (stomach, small intestine (duodenum, jejunum, ileum) or colon) (Fig. 7). Animal weight did not differ from the start to the end of the studies (data not shown). In addition, there was no indicative damage that could be seen in the liver or kidneys, and no exines were identified in any of these tissue sections.

## Discussion

This is the first study of its kind to undertake an *in vitro* assessment of differing SpEC manufacturing methods and to determine if the *in vitro* results are reflected in an acute oral *in vivo* response. The results from the *in vivo* assay of decreased ATP as an indicator of a reduction in cell viability showed that raw pollen had a cytotoxic effect, reducing the cell viability to below 90%, but did not show significant toxicity as defined by <50% of the cell control. Removal of the protein, pigments, carbohydrate and genetic material in SpEC preparations 1 (DES), 3 (BHS sporopollenin, hypochlorite bleached & sodium bromide), 5 (BHS, peroxide bleached) and 6 (AHS, hypochlorite bleached) increased the cell viability of these preparations to above the 90% acceptable cytotoxicity threshold and none of these SpEC preparations differed in cytotoxicity relative to the baseline cell control. This indicates that, potentially, either residual surface proteins and/or residual carbohydrates on the SpECs may be having a detrimental effect *in vitro*; however, as each of the methods will expose differing chemical functionalities peculiar to the sporopollenin fabric on the SpEC surface,^30^ including carboxylic acids, phenolics, lactones and polyhydroxylated aliphatics, that these may be affecting cell viability.

Not surprisingly, the raw *L. clavatum* spores were deleterious to cell viability; however, a relatively long and simple treatment (16 h) with 6% (w/v) sodium hydroxide solution resulted in good cell viability as in SpEC 1. By contrast, a shorter treatment (6 h) as in SpEC 2 gave poor cell viability, despite the additional hypochlorite treatment in the latter. Hypochlorite is known to be proteolytic^31^ but the damage appears to be insufficient to provide good cell viability as in the SpEC 1 formulation. Comparing SpEC 2 formulation with SpEC 3, both using the same concentration of hypochlorite, the latter employs bromide as a catalyst;^32^ hence, the additional activity of the oxidative combination appears to be sufficient to cause damage to residual proteins (undetectable, in our case, by combustion elemental analysis) or any other component on the SpEC surfaces resulting in SpEC 3 showing good cell viability. Perhaps not surprisingly, SpEC 4 formulation is very similar to SpEC 2 using a shorter treatment of 6% (w/v) sodium hydroxide solution than in SpEC 2; hence, again, such treatment is insufficient to damage such as residual proteins or any other component on the SpEC surfaces and to allow SpEC 4 to have good cell viability; furthermore, any residual SDS released from the SpEC 4 surface is likely to be deleterious to cells.^33^ These results indicate that the length of treatment for any given preparatory method needs to be optimised and this assay may help to determine what that optimum time is. BEAS-2B cells do not represent the gastrointestinal epithelium and were not intended to model the intestinal environment. Rather, BEAS-2B cells were used as a well-established, non-tumorigenic human epithelial cell line commonly employed in toxicological screening to provide a sensitive first-pass assessment of epithelial cytotoxicity across different SpEC preparations. Unlike intestinal epithelium, BEAS-2B lacks a mucus layer. Hence, any toxicity observed in BEAS-2B is therefore more stringent than expected *in vivo*. The use of BEAS-2B in our study is for comparative cytotoxicity, not for mechanistic gastrointestinal conclusions. We have the *in vitro* data as a screening filter, and rely on the *in vivo* data for biological relevance. Importantly, gastrointestinal relevance was addressed directly *in vivo* through oral gavage and comprehensive histological analysis of the stomach, small intestine, and colon, which showed no evidence of macrophage infiltration or tissue damage for any preparation. Future studies incorporating intestinal epithelial models (*e.g.* Caco-2, mucus-producing co-cultures, or organoids) would be valuable to further refine mechanistic insights.

Comparing SpEC 5 using hydrogen peroxide with SpEC 2 using hypochlorite, both involve the same level of sodium hydroxide pre-treatment, but the former allows good cell viability indicating hydrogen peroxide to be more beneficial than hypochlorite. In such a comparison, in this study it should be noted that the concentration (30% v/v) of peroxide was much higher than that of the hypochlorite (7% w/v), which may have been influential. However, to perhaps accord with this observation, it has been reported that hydrogen peroxide, in a dry-mist disinfection system, is significantly more effective than sodium hypochlorite solution at eradicating *C. difficile* spores.^34^

Harsh exine treatment preparation reflected in better cell viability as seen for preparations 1, 3, 5 and 6. Harsh treatment has been used to strip cytoplasm and the cellulosic intine of the *L. clavatum* spores to leave only the sporopollenin exine,^35^ removing any residual materials such as proteins or polysaccharides.

The mucus barrier in the gastrointestinal tract is composed of a thick, gel-like layer of mucus secreted by goblet cells in the epithelial lining, and protects the underlying epithelial cells and maintaining gut health.^36^ It acts as a physical barrier that can trap pathogens and particles and yet has selective permeability;^37^ therefore, it is likely that the SpECs do not come into contact with the gastrointestinal epithelial cells directly, and that the selective permeability of the mucus lining would allow an orally delivered active agent.

Combustion elemental analysis that showed 0% nitrogen detection suggests that all the protein was removed by each of the methods producing SpEC preparations 1, 3, 5 and 6, as these had no effect on cell viability. Conversely, the raw *L. clavatum* spores and SpEC 2 and 4 preparations (BHS, hypochlorite bleached and BHS, hypochlorite bleached with SDS) showed significant toxicity compared to the cell control, though the toxicity of SpECs 2 and 4 did not differ in cytotoxicity compared to the raw *L. clavatum* spores. Given that combustion elemental analysis showed 0% nitrogen detection in the SpEC 2 and 4 preparations as well, it is possible that the method was not sensitive to detect residual detrimental protein responsible for their increase in cell toxicity, or that the preparatory method was responsible.

In the *in vivo* experiment, there were no differences between any of the preparations in the white cell count, lymphocyte count, monocyte count or the neutrophil count indicating no overt inflammatory response. There were no differences between any of the preparations in the histological specimens of the entire gastrointestinal tract, and the lack of macrophage infiltration in the gastrointestinal tract is particularly reassuring indicating that an immune response was not being provoked in the acute setting. CD68 positivity, which indicates the presence of macrophages, typically appears in tissues within 24 to 48 hours after an acute injury, with robust, significant infiltration usually observed by 5–7 days;^38,39^ therefore, CD68 positivity within the 5 day time course would have given a robust response to an acute injury. Thus, SpEC preparation 4, whilst showing significant toxicity *in vitro*, did not cause any intestinal irritation or inflammation, and neither did the raw *L. clavatum* spores. CD68, a lysosomal glycoprotein, was specifically chosen for immunostaining as it is expressed by monocytes and macrophages and would be indicative of significant tissue inflammation with cellular infiltration, and that was not found here for any exine preparation in the *in vivo* study. It is therefore likely that, at least in the acute setting and in the presence of an intact gastrointestinal mucosa, that the SpECs produced by any method will be safe to ingest and not cause irritation although, from our results, it would be preferable to ingest SpEC preparations 1, 3, 5 or 6 in comparison to SpEC preparations 2 and 4. There is evidence to demonstrate that mucoadhesion is playing a role towards the observed high (10×) enhanced bioavailability, such as found with eicosapentaenoic acid^14^ and vitamin D,^26^ with measurement of exine mucoadhesion.^26^ Unique features of SpECs are their topography, chemistry that is particular to sporopollenin and choice of capsule size. These characteristics are peculiar to the SpECs' plant origin. The SpECs are too large to pass through the gut wall to explain the high (10 ×) enhanced bioavailability. It is likely that mucoadhesion or bioadhesion is the major role in the mechanism of drug delivery in the gastrointestinal tract as we have shown previously.^26^ In support of this, physical^18^ and chemical^11,16,19,40^ properties of the outer surface of SpECs would appear to match those that are required for bioadhesion. SpECs are amphiphilic, possessing a cross linking of hydrophobic saturated and unsaturated carbon chains that have functional groups attached, including phenyls, phenols, aliphatic alcohols and carboxylic acids that are capable of hydrogen bonding. Furthermore, it is known that pollen-stigma adhesion often happens by a species-specific hydrophilic attraction.^41^ It may have been expected that the mucoadhesion would potentiate any local cellular damage but this would not appear to be the case and that there is no damage is additional evidence that the SpECs do not traverse the gastrointestinal wall when damage would have been expected to be seen and this aligns with other reports.^26^ In a study delivering the nonsteroidal anti-inflammatory preparation diclofenac,^42^ SpECs were reported in the bloodstream of the animals; however, this may have resulted from the diclofenac that can cause considerable gastric irritation and bleeding, thus broaching the gastrointestinal barrier and then may have been enhanced by the cytotoxic effect of the exine preparation used for the diclofenac delivery. Alternatively, SpECs could have contaminated the samples under investigation in an environment where SpECs or spores have been used. In this study, no SpECs were observed in the bloodstream or organs in this study. This indicates that the exines loaded with a pharmaceutical agent would be excellent at protecting against the harsh stomach environment with release of the payload in the intestine, without intestinal irritation by the SpECs and without the absorption of the exines that are then fecally excreted.

The strength of this study is that it fills a gap in the existing literature by providing a comparative analysis of different SpEC preparations. Limitations of this study include that combustion elemental analysis and FTIR were used to determine if the SpEC preparations were protein free, and it would be ideally necessary to perform mass spectrometry^17^ to confirm that with any samples being taken forward. This was an acute study, and it would be necessary to repeat it chronically to see if any allergic response occurred upon ingestion of raw *L. clavatum* spores that was ameliorated or prevented by the prepared SpEC preparations over a period of a month, for example. The BEAS-2B epithelial cells to assess cytotoxicity are commonly used in toxicological studies, and it would be worth exploring the cytotoxic effects on other tissue derived cells lines. Future studies on those SpEC preparations will require detailed and time-intensive residue and contaminant analysis.

In conclusion, whilst differing SpEC manufacturing processes may have residual *in vitro* cytotoxicity, this did not translate to an acute *in vivo* oral gastrointestinal immune response, suggesting their safety as a vehicle for gastrointestinal pharmaceutical delivery. However, longer term *in vivo* data is needed to confirm they are immune-inert following prolonged oral ingestion.

## Ethics approval and consent to participate

All animal protocols were approved by the Ethical Committee for Animal Experiments of Arabian Gulf University (reference number E29-PI-2-23).

## Consent for publication

All authors gave their consent for publication.

## Author contributions

Conceptualization, SLA, AEB and KG; methodology, AH, JS, ASMM, HB, SW and MA; investigation, AH, JS, ASMM, HB, SW, MA, PM and RD.; writing – original draft preparation, SLA and AEB; writing – review and editing, AH, JS, ASMM, HB, SW, MA, KG, ANB, PM, RD, SLA and AEB; visualization, AH, JS, ASMM, HB, SW, PM and RD. All authors have read and agreed to the published version of the manuscript.

## Conflicts of interest

No authors have any competing interests to declare, financial or otherwise.

## Supplementary Material

RA-016-D5RA09999D-s001

RA-016-D5RA09999D-s002

## Data Availability

All data will be made available upon reasonable request to the corresponding author. Supplementary information (SI) is available. See DOI: https://doi.org/10.1039/d5ra09999d.
