# Supplementary figures and images for: In vitro and in vivo assessment of sporopollenin exine capsule preparations (SpECs) from Lycopodium clavatum spores
Source: RSC Adv. 2026 Apr 7;16(20):17848–59. doi: 10.1039/d5ra09999d (PMC13054223; doi:10.1039/d5ra09999d)

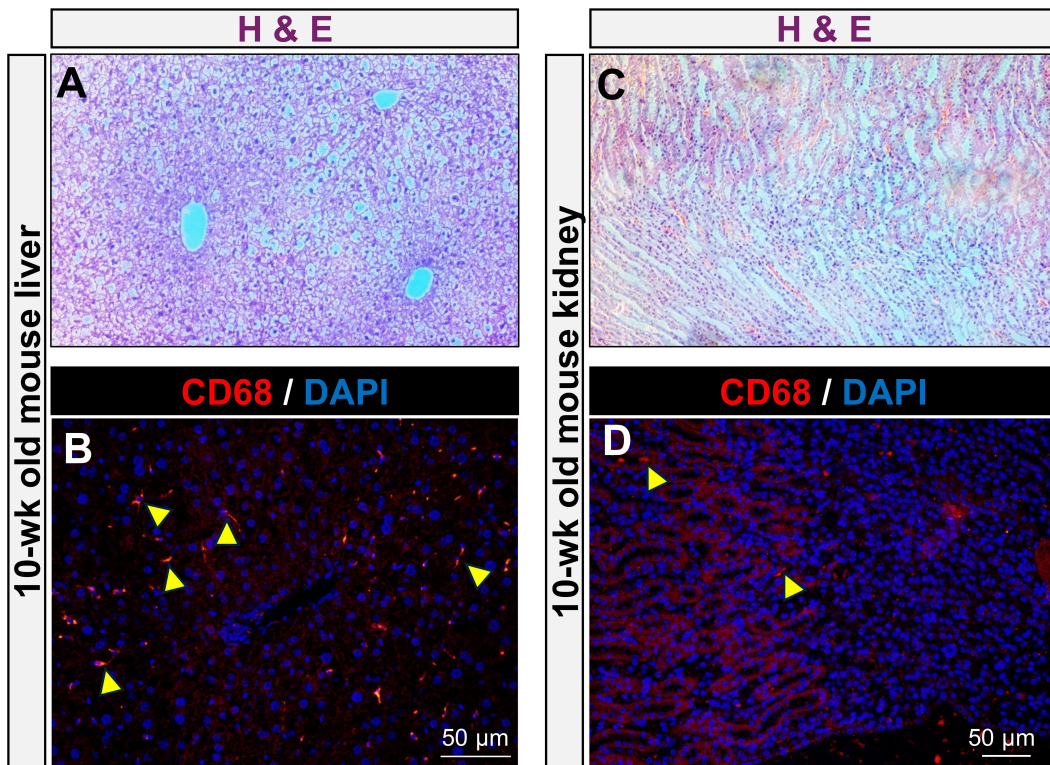

Supplement: RA-016-D5RA09999D-s002 [file RA-016-D5RA09999D-s002.pdf]
